# The influence that Spanish Labour Reform represents on Madrid Stock Market: An empirical analysis

**DOI:** 10.1371/journal.pone.0258004

**Published:** 2021-10-06

**Authors:** Ana M. Sabater Marcos, Teresa Duarte Atoche, Joaquina Laffarga Briones

**Affiliations:** 1 Universidad Miguel Hernández, Alacant, Spain; 2 Universidad de Sevilla, Seville, Spain; University of Almeria, SPAIN

## Abstract

Empirical evidence for Spanish Stock Market shows that labour events, like a firm level collective agreement, have informative content for the market due to the loss of wealth that it implies for the investor. Labour Reforms which Spain experienced between the years 2010 and 2012 have allowed the jeopardising of employment and the destruction of jobs, substituting one well paid by another of lower cost for the firm, the cost of dismissal, or the proposals of substituting payoffs by the so-called Austrian backpack, and the elimination of the distinction between temporary and permanent contracts. These Labour Reforms affect many of the accounting and financial variables, which are the subject of analysis and follow-up by investors and analysts, next to the idiosyncrasy of the Open Shop System that is followed in Spain, the present article means to explore the effect on Madrid Stock Market. Our results, applying analysis techniques with decision trees where we control the effect of the economic crisis on the market reaction, show that the Labour Reforms of 2010 to 2012 are incorporated as negative, or positive, information when the investor perceives a possible decrease, or increase, in its future cash flows.

## 1. Introduction

The regulation of labour relations and the work market in Spain have experienced significant reforms since of the creation of the Workers’ Statute in 1980. These reforms also tend to be greater and more thorough in periods of economic crisis (as is the case of the reforms of 2010, 2011 and 2012) than in periods of expansion. The main reason for this is the worse work market situation in those periods of economic crisis and the greater pressures existing to improve that situation. This is especially so when the institutions dominated by economic orthodoxy have been denouncing the rigidity of the labour market for years, blaming it for artificially maintaining a high work cost.

Neoclassical theory considers work like any other resource which contributes to the production process. If the supply surpasses the demand, it will be enough to lower the salary to return to the market equilibrium. Furthermore, a lower salary also causes less offer as there are workers who are not willing to work at this price. Following this theory, unemployment is voluntary as the only thing that has to be done to eradicate it is to work at the price at which firms are willing to contract all the work available in the market. Therefore, if salaries do not drop it is because of trade unions and the regulation which protects the workers’ rights, or else the lack of the regulations’ flexibility prevent the market from adjusting.

It is exactly at this point that the labour reforms arose in 2010, 2011 and 2012. These have allowed the jeopardising of employment and the destruction of jobs, substituting one well paid by another of lower cost for the firm. Not to mention the cost of dismissal, or the proposals of substituting payoffs by the so-called Austrian backpack, and the elimination of the distinction between temporary and permanent contracts.

Moreover, these growing demands for new labour reforms are stimulated and reinforced by the substantial shift which took place in economic policy in May 2010, by suppressing Keynesian economic and tax stimuli which had been established in spring 2009 and substituting them by a strict fiscal austerity policy. This means that the prior and fundamental aim of economic policy is the reduction of the deficit and of the public debt. The objectives of the creation of employment and the reduction of unemployment remain in the background, subordinated in any case to the success of the economic policy of austerity in the improvement and the reestablishing of market confidence in the Spanish economy. But how did the Spanish financial market really interpret the different labour reforms?

Collective bargaining is an aspect which analysts and investors take into account due to the multitude of scenarios which, in one way or another, affect the firm’s future cash flows and that, thereby, modify the distribution of wealth. The empirical evidence for the Spanish market demonstrates that the signing of a firm’s collective agreement is interpreted as bad news by the investor, due to the increase in labour costs which it involves.

There are a number of papers addressing relationships between collective bargaining and corporate performance in a financial context. These works have been widely focused on the Anglo-American context. For instance, [1], and [2] found that firms’ stock prices fell for the US case in the presence of unionisation within a firm. In the same line, [3] observes that shocks in labour costs cause a proportional decrease in a firm’s stock price. From a slightly different approach, [4] measures corporative performance by using Tobin’s Q and finds a negative correlation between union presence and corporative performance. Moreover, [5] found that firms with high union power have reduced stock prices and R&D investments. Moreover, [6] found that collective bargaining affects distribution, but it does not have an impact on production or on the use of productive factors. For Europe, several papers study the relationship between stock performance and collective bargaining. [7–10], who focus on the Spanish context, found that the relationship between collective bargaining and stock performance is negative around the date of the event.

A collective agreement is a written agreement, negotiated between the workers and the management, which establishes the workers’ labour and economic conditions. The agreements most used in Spain are sectorial agreements and firm agreements. Sectorial agreements establish the minimum salary of the workers of a specific sector. The firm, for its part, can voluntarily disassociate itself from the sectorial agreement and improve its conditions in an own or firm agreement. This will depend on the power and the strength that the trade union has in the company, as well as its financial capacity.

There exist two models of collective bargaining, the Open Shop typical of European countries and the Closed Shop typical of Anglo-Saxon countries. The Open Shop System in Spain, is characterised by the result of the bargaining affecting all the workers regardless of their being affiliated to a trade union, unlike the USA, for example, where the result is applied only to unionised workers; hence the unionisation rate in Spain is very low compared with the USA or Canada.

Actually, there has been some international evidence to suggest that the existence of a trade union and potentially a collective agreement have statistically and economically significant positive effects on the productivity of the firm [11].

While, there has been prior literature to support such sequence based mainly in the US, there are international studies presenting evidence that there is no uniform reaction in all countries. [12], for example, examined the effects of unions on firm productivity and financial performance in different national settings. He observed a negative relationship for the case of the US, as in previous literature, however in the UK the adverse effect is reversed in recent years, while in Germany and Japan unions (and apparently collective bargaining) have a positive effect on productivity and financial performance. In countries with stronger trade unions, high unionisation and considerably often industrial action, like in continental Europe (on union strength in EU see for example the collective bargaining coverage at [13], failure to reach a collective agreement may lead to prolonged strikes, social unrest, employee dissatisfaction, a potential fall in productivity and a decline in share prices (e.g. [14], on the impact of strikes on stock value).

[10] observed, through a theoretical model for the Madrid Stock Market, which risk adverse investors modify their portfolios when faced with the signing of a collective firm agreement and this result is maintained even in those companies that have stipulated an increase in productivity in their agreement. So, the institutional characteristics of each country will play a decisive role in the results obtained.

The present article means to explore the effect of the Labour Reforms which Spain experienced between the years 2010 and 2012 on the share prices of the Madrid Stock Market. Among the numerous reforms introduced is the flexibility granted to firms to disengage from the agreement and reduce the workers’ salaries, and the prevalence of firm agreements compared to sectorial agreements.

Given that the Labour Reforms directly affect many of the accounting and financial variables which are the subject of analysis and follow-up by investors and analysts, we propose the hypotheses of our work supposing that risk-adverse investors manage their value portfolio taking labour relations into account. The Labour Reforms of 2010 to 2012 are incorporated as negative information, or positive, when the variables affected involve a decrease, or increase, in its future cash flows. Our results, applying analysis techniques with decision trees, where we control the effect of the economic crisis on the market reaction, endorse our hypotheses.

The work is structured as follows. The labour reforms passed during the economic crisis are mentioned in detail. The sample and the methodology are presented. The results and sensitivity analysis are set out, and the conclusions are drawn.

## 2. The labour reforms passed during the economic crisis

The labour reforms whose effects on the accounts of firm results and, thereby, on their profitability, are going to be analysed in this article are those which were carried out in the years after the outbreak of the crisis. This crisis began with the banking crash derived from the massive diffusion of products stemming from subprime mortgages in the United States. More specifically, the reforms which were carried out through the following legal texts are:

- Royal Decree-Law 10/2010, of 16 June, of urgent measures for the labour reform of the work market, which establishes measures that affect numerous matters of labour regulation with three main aims: a) to reduce the duality of the labour market; b) to reinforce the instruments of internal flexibility in the development of labour relations and, in particular, the measures of temporary reduced working hours and c) to raise the opportunities of unemployed people, paying particular attention to young people, reordering the bonus policy to do so.
- Royal Decree-Law 7/2011, of 10 June, of urgent measures for the reform of collective bargaining which will substantially modify the structure, competition, content, effect, legitimacy, processing, application and interpretation of collective agreements.
- Royal Decree-Law 3/2012, of 10 February, of urgent measures for the reform of the labour market -possibly the most far-reaching legal reform since the passing of the Statute of Workers of 1980 as the legal changes which it introduced substantially altered the basic principles which, until that moment, had sustained the Spanish labour system in the area of individual and collective behaviours in the labour process and in the systems of protection. This meant a substantial and substantive modification of the rules of the game existing till then.

The presenting of the motives of these three legal texts which enacted them assumed two main principles or starting points. On the one hand, which to recuperate the path of economic growth and to detain the enormous destruction of employment which the recession brought, it was indispensable to intervene as a matter of priority in the labour market. On the other hand, the nature of the predominant labour model at that moment was incompatible with the recovery and emerging from the crisis, so it was necessary to go much more intensely into the lines which had followed the labour reforms carried out before. These are all of inspired in the idea that the Spanish labour market is extremely rigid and that, therefore, it is necessary to increase its flexibility, enabling a more appropriate adjustment between the supply and the demand of work. In the opinion of the legislators this is the only way to achieve a reduction of employment via generating lower balanced salaries and increase productivity.

To this end, the reforms mentioned proposed three fundamental objectives and a basic procedure to attain them. This would be defined by the singular effectiveness of the last reform of 2012.

These aims were to gain the greatest possible flexibility in all the labour relations, to reduce the segmentation of the market, favouring the rotation and mobility of the work force and to improve the workers’ conditions of employability. And the basic procedure which should permit achieving them would be the modification of the bargaining procedures, more specifically the degree of centralisation of the collective bargaining system and the redundancy costs and procedure.

Wage devaluation could be ultimately attained thanks to establishing a new balance of powers of decision. That is to say, a decrease of the balance price -of the salary- which, according to the dominant economic theory, is the essential condition for an appropriate adjustment to be produced, and employment tending to total employment between the supply and demand of work.

We next present the most specific measures which were passed in these three legal reforms to later substantiate their real effects on business behaviour.

### Urgent measures for the labour reform of the work market of 2010 (R.D-L. 10/2010)

In this first reform the following main reforms can be distinguished.

- Temporary contracting. Two novelties are introduced. The first consists of the establishing in the Workers Statute of a time limitation of the contracts for a three-year specific work or service, extendable twelve months more if this is included in the collective bargaining, which prevents their perpetuation. The second refers to the payoffs before finalising the contracts for a specific work or service, which was 8 days per year worked and is established as 12 days per year worked, a transitional regime being established.
- Redundancy, individual or collective termination of work for objective reasons. Four main questions are adopted. The first refers to the notice period for current losses or those foreseen that the Workers Statute maintained at thirty days and which the reform reduces to fifteen days. The second is a technical question, but one of unquestionable practical significance, which eliminates the suppression of nullity on formal grounds. The third is the assuming by the Wages Guarantee Fund of the amount of eight days per year worked of the redundancy, presuming termination for objective causes. The fourth, the possibility of redundancy for absenteeism of those workers who are not at work for more than 20% of the working days over two consecutive months, or 25% if these are four discontinuous months within a period of 12 months, whenever the average of labour absenteeism in the firm surpasses 2.5%.
- Collective agreements. The possibility of not applying the salary regime established in a collective agreement of a wider scope than the firm’s is considered if this is in crisis.
- Workday. The possibility of reducing the workday up to 70% with the consequent salary reduction for economic causes and with the right to unemployment pay for the hours affected.
- Labour intermediation and temporary work firms. The figure of profit-seeking placement agencies is regulated. These will act along with the public employment service. The restrictions to which temporary work firms were subject to operate in specific sectors are revised and the contracts for training are enhanced, with the aim of facilitating access to work of young people, both by bonuses and improving their conditions.

### Urgent measures for the reform of collective bargaining of 2011 (R.D-L. 7/2011)

The modification of the collective bargaining system is produced via changes in the following main aspects:

- The structure and competition of collective bargaining. The prevalence of the firm collective agreement over the sectorial collective agreement of any area is facilitated, except the autonomous or statewide collective agreement. The possibility of competing with a statewide sectorial collective agreement is limited to the autonomous sectorial collective agreement, provincial agreements now not being included.
- The content and validity of collective agreements. A minimum period to denounce the collective agreement and the maximum periods for the beginning and the negotiation of the new collective agreement are established. There is also an obligation to incorporate measures of internal flexibility in the agreements. Binding arbitration in the cases of a lack of agreement are incorporated as a subsidiary regulation.
- The legitimisation for the negotiation of collective agreements. The role of the trade union sections is reinforced as is their priority over works councils.
- Internal flexibility. Prevalence in the negotiation of the trade union sections over unitary representation and the introduction of the «persistent decrease of the level of incomes» as a cause allows the non-application of the salary regime foreseen in the collective agreements of a wider scope than the firm. A possible disagreement with regard to the Joint commission of the agreement is put forward. This will have a period of seven days to give its opinion, counting from the discrepancy which was proposed.

### Urgent measures for the reform of the labour market of 2012 (R.D-L 3/2012)

This reform included a broad set of modifications in the institutions of the work market which, as has been said, meant the most radical change of nature of the Spanish system of labour relations during the last thirty years. Its main changes are the following:

- Collective bargaining. The applicative priority of firm agreements in an ample set of matters is established (basic salary, supplements, complements, pay for overtime or distribution of work time). This implies that these agreements can modify what is foreseen in agreements of a wider scope without any kind of limitation. Likewise, the possibilities of cuts in pay and in the rest of the work conditions are facilitated, in the case of redundancies the causes which permit the cuts being eased. Furthermore, in the case of a cut or the non-application of an agreement in force, although this must be produced in agreement with the workers, if such an agreement is not reached, upon request of any of the parties (normally this will be the firm) arbitrage can take place. Finally, ultractivity is eliminated (which means the extension of the agreements that have finalised their period of validity) when a year has passed since the agreement had been denounced, the validity of the agreement lapsing in this case and the workers remaining without an agreement when there does not exist another agreement of a wider scope of application.
- Work conditions. The reform introduces a very considerable change: the possibility of modifying the work conditions (including wages), when these conditions, including the wages, are not established in a collective agreement but, for example, in an individual contract or via a collective agreement or pact different from the statutory collective agreement. It is established that to do so it will be sufficient for the need of modifying the work conditions to be generally related with competitiveness, productivity or technical organisation or of work in the firm, without it being necessary (as established in the previous regulation) for the modifications to contribute to foreseeing a negative evolution of the firm or to improving its situation or perspectives.
- Redundancy cost and procedure. On the one hand, it reduces the compensation for unfair dismissal from 45 days of wages per year of service with a limit of 42 monthly payments to 33 days with a limit of 24 monthly payments, thus considerably decreasing the difference between the cost of unfair dismissal, without cause, and fair dismissal for objective causes. On the other hand, the causes for objective dismissal are very substantially eased, especially those which are economic, as is permitted when the firms can present losses, or simply expect them or experience an interannual reduction of the ordinary incomes or sales during three consecutive quarters. And, finally, the obligation of the administrative authorisation for collective dismissals and the payment by the firm of the procedural salaries between the moment of notification of the dismissal and its court decision is eliminated.
- Contracting. A new type of contact, in principal indefinite, is introduced for firms with less than 50 workers, but with a test period extended to a year, during which the worker can be freely dismissed without fiscal bonuses being lost. On the other hand, the contract for training and learning is eased, allowing successive contracts of this type for the same worker by the same or another firm, as well as a part-time contract, doing additional overtime to the existing extra hours being authorised.

The study of the impact of these three reforms on Madrid Stock Market will be the aim of the rest of the article.

## 3. Sample and methodology

Our sample covers firms quoted on Madrid Stock Market between January 2 2010 and December 31 2012. We proceeded as follows. First, we obtained the firms quoted on Madrid Stock Market from the website. We consider the date on which the labour reform was published in the BOE as the zero moment. We check that this is correct by conducting a data search in the economic press and the website of the National Stock Market Commission (CNMV). We found that the announcement of the reform was published on the very day that the labour reform was published.

Second, we selected the length of the event window in order to test abnormal behaviour in the returns of the sample firms. Although most information is usually quickly incorporated into stock prices, some information may sometimes leak out before formal publication, or publication may be delayed. Then, we considered five days before and after the zero moment.

Firms which happen to have more than one relevant announcement within the event window (collective agreements, mergers, splits, dividend announcements…) were excluded from the sample to avoid any potential confusing effects. The remaining sample after these exclusions, consisted of 61 firms for 2010, 70 firms for 2011 and 53 firms in 2012 for 30 sectors according to the two-digit sector classification. The industries are: trade and other services, other manufacturing industries, cement, glass and construction materials, finance, transport and communications, utilities and construction, among others.

In addition, we generated two subsamples, one composed of companies only with a firm-level agreement and another subsample made up of companies with sector agreement.

We work with three event dates, corresponding to the three labour reforms under study:

Reform 2010. Royal Decree-Law 10/2010, of June 16, on urgent measures for the reform of the labour market. BOE 06/17/2010
Reform 2011. Royal Decree-Law 7/2011, of June 10, on urgent measures for the reform of collective bargaining. BOE 06/11/2011
Reform 2012. Royal Decree-Law 3/2012, of February 10, on urgent measures for the reform of the labour market. BOE 02/11/2012

The distribution of the sample among sectors and years is illustrated in S1 Table. Among the sectors, the financial stands out, where there is a greater number of companies in both subsamples. In the construction sector, the largest number of listed companies under the sectorial agreement is highlighted, as well as the civil engineering sector. With regard to the subsample of companies with their own agreement, the bulk of the sample is found among finance companies, metallurgy, manufacture of machinery and other materials, radio and television broadcasting, and consulting activities. The information was drawn from the Thomson Database.

As already mentioned in the introduction, the aim is to explore whether the labour reform has an impact on a firm’s stock price. To this purpose Event Study technique is used. For further information on the Event Study methodology see [15].

Since stock prices reflect the true value of a company and change immediately in response to any event that may potentially affect the company’s future cashflows, the impact on the corporate value of a given event can be measured by observing stock price changes over a very short time period around the date of the event. The variable is the occurrence of abnormal returns in companies around the date of the event. In order to calculate this, the return given by the market model as normal will be used.


$$R_{it}=\alpha_{i}+\beta_{i}R_{mt}+\varepsilon_{it}$$ (1)

Where R_it_ is the return on company *i* on day *t*; R_mt_ is the return on the market portfolio on day t; α_i_ is the expected return on company *i*, which is independent from the market; β_i_ is the sensitivity of the return on company *i* to changes in market return; and ε_it_ is a random perturbation. The market portfolio is represented by the IBEX35 index.

As occurs in most studies on events that affect several companies on the same date, there is an overlap of events in the estimation periods, so it must be taken into account that the residuals ε_it_ are not independent but are correlated. In addition, a frequent heteroscedasticity problem can arise in cross-sectional analyses. An appropriate regression model when considering heteroscedasticity and the contemporary correlation of residuals is the Seemingly Unrelated Regressions (SUR) model. Therefore, it is important to know if there is a contemporary correlation, since if there is no separate OLS for each equation, it is as efficient as SUR. See [16]. Applying the Breusch-Pagan test we obtain that there is a contemporary correlation. This model proposes that the correlation of the contemporary residuals is different from zero and the non-contemporary residuals are equal to zero.

Therefore, instead of estimating the classic market model for each event by OLS, we will use the system of Eq (2), estimating the coefficients by GLS. This will allow us to have better estimation results, taking into account temporal correlations and cross-section heteroscedasticity.

$$R_{1t}=\alpha_{1}+\beta_{1}R_{\mathrm{mt}}+\varepsilon_{1t}$$

$$R_{2t}=\alpha_{2}+\beta_{2}R_{mt}+\varepsilon_{2t}$$ (2)

$$R_{\mathrm{nt}}=\alpha_{n}+\beta_{n}R_{\mathrm{mt}}+\varepsilon_{\mathrm{nt}}$$

Estimating this system of equations enables calculating the daily abnormal returns (ARit) for a news item from company i:

$$AR_{it}=R_{it}‐(a_{i}+b_{i}R_{mt})$$ (3)

Where *a_i_* and *b*_i_ are the GLS estimates obtained in the regressions [2] by using a period of 145 days before the announcement. This is an appropriate period of time for estimating the parameters according to available empirical evidence on event study [9]. Abnormal returns from stocks are averaged in a cross section throughout each day of the event window or study window, producing the average daily abnormal returns AR_t_

Considering that the market may anticipate information regarding the event or that delays may occur in its announcement, there is an event period of 11 days around the date that the labour reform is published: from day T_1_ = -5 to day T_2_ = +5. For a more comprehensive analysis, the cumulative abnormal returns *CAR* (*t_1_, t_2_*) were calculated in order to find out the cumulative effect of the event.

If the labour reform conveys new information to investors, the expected value of the abnormal returns must be significantly different from zero. In order to test this hypothesis, the bootstrap technique was used. An analysis of the evolution of abnormal returns in the study window indicates that some of the distributions are slightly biased and present leptokurtosis. The Jarque-Bera test does not validate the normal distribution of the sample and, therefore, the proposed hypothesis must be tested using a non-parametric test. This study likewise incorporates a non-parametric test based on the bootstrap methodology. The test aims at obtaining the empirical distribution of the target variable and testing its significance based on the simulated distribution. The distribution of the conventional t statistic is simulated in order to obtain critical values from the simulated distribution.

Next, how a labour reform may lead to changes of a firm’s share price was empirically investigated, controlling the possible effects that the country’s macroeconomic situation may have, such as the Risk Premium (PR), the Slope of the Sovereign Yield Curve (PC) and the Ted Spread (TED), different macrovariables that we introduce as control variables.


$$R_{it}=\alpha_{i}+\beta_{1i}R_{mt}+\beta_{2i}PR_{}+\beta_{3i}PC_{}+\beta_{4i}TED+\varepsilon_{it}$$ (4)

Where R_it_ is the return on company *i* on day t; R_mt_ is the return on the market (IBEX35) on day t; α_i_ is the expected return of company *i* that is independent of the market; β_1i_ is the sensitivity of the return on company *i* to variations in market return; β_2.3,4 i_ is the sensitivity of the return on company *i* to PR, PC and TED respectively, and ε_it_ is a random perturbation.

### Spanish Risk Premium (PR)

Internal Rate of Return (IRR) Spanish 10 years bond–IRR German 10 years bond or like ratio (IRR Spanish 10 years bond/IRR German 10 years bond)

### Slope of the Sovereign Yield Curve (PC)

Internal Rate of Return (IRR) Spanish 10 years bond–interest rate three-month Spanish Treasury bill or like ratio.

### Ted Spread (TED)

The difference between the three-month Treasury bill and the three-month LIBOR based on US dollars. In other words, the TED spread is the difference between the interest rate on short-term government debt in the United States and the interest rate on interbank loans.

In this way, it was checked whether any effect or reaction that arises in the Market is due to the macroeconomics circumstances of the Market and not to the event itself.

An analysis of covariance between the different variables, see S2 Table, shows us the correlation between them, which leads us to carry out different models incorporating in each one of them the different variables created to control the effect of the economic situation.

Next, we calculate the abnormal returns for each day of the event window and accumulated in different analysis windows.

In S3 Table, we can see daily abnormal returns for each day of the event window for our three reforms, controlling the possible effects that the country´s macroeconomic situation may have using the PR, PC and TED variables.

The first panel shows the results for the 2010 reform. These results indicate that this event has informative content for the market as there are daily abnormal returns significantly different from zero. The results are mixed, if we observe the abnormal daily returns of the market model without controlling the effect that the economic situation of that year could have, we note an erratic behavior in the event window. There are significantly non-zero negative abnormal returns on day -4, and day +1, and significant positive returns for bootstrap on days -2, +2, and +4.

If we see the daily abnormal returns for the 2010 reform, incorporating the macro variables into the market model, we observe a more uniform behavior throughout the days when the presence of positive abnormal returns significantly different from zero for bootstrap prevails, at 5% and 1%, on days -2, +2, +3 and +4 with positive abnormal returns around + 0.8%.

Next, in the second panel, we observe the results for the 2011 reform. In this event the results are similar regardless of whether we control for the effect that the macro situation may have on the results. We see significant negative abnormal returns for days –5, -2, -1, for the zero moment, and also on days +3, +4 and +5 after the publication of the 2011 reform. Negative abnormal returns are, on average, around -0.8%, with a level of significance between 5% and 1%.

The third panel shows the daily abnormal returns in the event window for the 2012 reform. With the exception of day -5, which indicates positive abnormal returns around + 0.4%, in the rest of the days of the event window there are no significant returns in any of the models under study.

S4 Table shows the cumulated abnormal returns through different windows to determine the accumulated effect of the event.

The first panel shows the results for the 2010 reform. Again, if we only apply the market model in calculating returns, the results are mixed. If we control the effect of the macroeconomic situation, we can observe more uniform and conclusive results. All study windows, with the exception of window (-1, + 1), show positive cumulative abnormal returns significantly different from zero for bootstrap. In particular, in the event window we observe a significant accumulated average abnormal return of + 2.94%, + 4.58% and + 2.85% when we use the slope of the yield curve, the risk premium and the Ted Spread as a control variable, respectively. This result is maintained in the windows (-3, + 3) and (-2, +2), as well as in the pre-event (-5, -1) and post-event (+ 1, + 5) windows.

The 2010 reform is interpreted as good news by the market as shown by positive abnormal returns. This result is more evident when we control the effect of the crisis through macro variables This first reform of the labour market in Spain represented a turning point in the conditions that companies had to meet in order to carry out massive layoffs, as well as a reduction in the cost of dismissal and a clear salary drop for companies covered by the sectorial agreement. If the Spanish market in recent years has interpreted the signing of a company agreement as bad news due to the decrease in future cash flow that it implies, it is to be expected that the market will reward a reduction in labour costs as a result of the 2010 reform. This result is in line with Neoclassical theory that considers work like any other resource which contributes to the production process. If the supply surpasses the demand, it will be enough to lower the salary to return to the market equilibrium so unemployment would be lower.

The second panel of S4 Table shows the results of the cumulated abnormal returns for the 2011 reform. These are observed in all the models and windows, the result being more forceful when macro variables are incorporated, resulting in the presence of significant different negative abnormal returns for bootstrap. In the event window, a cumulative average abnormal return of -5% significant to 1% is observed in all the models analysed.

Based on these results, the 2011 reform is interpreted as bad news by the market as shown by the significant negative abnormal returns in all the study windows. In the 2011 reform, the first changes in collective bargaining began, the most important being the prevalence of the firm level collective agreement over the sectorial agreement. In the sample analysed, the vast majority of companies are covered by the sectorial agreement as shown in S1 Table.

Previous empirical evidence for the Spanish market has shown that the signing of a company agreement is always interpreted as bad news and also has a spillover effect in companies with a sectorial agreement.

An increase in labour costs means lower accounting profit and lower future cash flows, with the consequent loss of wealth for the investor. The result is the consequence of the particularities of the collective bargaining system in Spain, where the results of the agreement are applied to all workers regardless of whether they are members of the union, the threat of a strike in the company and the presence of the company committees in the negotiation of the agreement are factors that make the loss of power of the unions more than unlikely.

In the setting under discussion, Spain, although the trade union density is not much higher than in the US ([17] OECD: 13.6% to 10.1% in 2018), the bargaining power of the unions is significantly higher, since due to the open-shop bargaining the collective agreements signed cover the majority of the employees (OECD: 80.1% for Spain to 11.7% for the US in 2018). In addition, industrial action is considerably often in Spain, although it has been reduced in the recent years. In a study by the European Trade Union Institute [18], Spain is the sixth out of 22 EU countries with the higher number of days not worked due to industrial action for the period 2010–2017. Therefore, the institutional characteristics of the collective bargaining system in Spain are decisive given the forcefulness shown by these results.

This change introduced in the reform may generate distrust in investors due to the risk that their companies dissociate themselves from the sectorial agreement and sign a firm level agreement, with the increase in labour costs that this would imply. In a subsequent analysis, we analyse whether the reform is interpreted differently depending on what type of agreement the company has.

Another explanation for this result can be found in the Paradox of Costs and in the post Keynesian employment curve. After the experience of the 2010 reform, employment continued to be destroyed in 2011 and the fall in labor costs that led to the reform in 2010 did not generate employment or wealth, so you could expect the market to interpret lower future cash flows due to the precariousness of working conditions and contracts.

In the time period of the 2011 reform, the international and national extreme (macro) economic developments, during the event-window, led to a confined crash in the Spanish Stock Market (the IBEX-35 declined by 3.6% during this period). During those days, the Spanish economy was entangled in the European sovereign debt crisis, the Spanish debt risk premium was climbing, the European Commission was asking for a series of hard measures for the Spanish economy and the stock market was facing an exit of investors, decline in the indices and cancelling or postponing of IPOs [19]. The sharp decline in share prices, with the subsequent herd behaviour by investors, led to high negative abnormal returns.

Panel 3 of S4 Table shows the results for the 2012 reform. If we look at the different models, no significant abnormal returns appear in any study window. In terms of working conditions, the most notable change introduced by the 2012 reform is that when these conditions, including wages, are not established in a collective agreement but, for example, in an individual contract or by means of a collective agreement other than the statutory collective agreement, this can be modified by a unilateral decision of the employer, the need to modify working conditions being generally related to competitiveness, productivity or the technical or work organisation of the company being sufficient, without (as it was in the previous regulations) modifications contributing to anticipating a negative evolution of the company or to improving its situation and perspectives being necessary.

It has been argued in prior literature that as a result of the Labour Reform in 2012 in terms of collective agreements and wages, the trade unions were led to a position of defensive bargaining while the attitude of firms at the bargaining table was changed “*from trying to give as little as possible to trying to obtain as much as possible*” [20]. In addition, since in the following years after the reform (2013–2014), the great majority of Spanish firms (81,95%) declared themselves quite or very satisfied with their collective agreement–although this includes old and new [20]. As it was described, Spain is in the top-6 countries in EU in terms of industrial action and prior literature documents an adverse relation between strikes and productivity. The 2012 Reform was the most radical in 30 years and the trade unions replied accordingly. In particular, “*the announcement of the reform immediately triggered a reaction from the trade unions*, *which called demonstrations across Spain on 18 and 19 February*. *Attendance at the protests was very significant*, *which the trade unions described as a show of ‘massive support’ from the people*, *and they announced an escalation of the mobilisation if the government did not improve its offer*” [21]. These events, as well as the announcement of a general strike, occurred within the event window of the study and it can be argued that they would have an effect to investors, creating uncertainty in the market and contributing to potentially contradicting observations that led to the statistically insignificant result.

We now see in S5 Table the evolution of abnormal returns, calculated with the market model and the slope of the interest rate curve (PC), by subsamples built based on whether the company has a firm level or sectorial agreement.

Regarding the 2010 reform, we remember that the market reacts positively to the arrival of new information on the labour reform, if we observe the behavior of the titles depending on the type of agreement we see that the sub-sample of companies with a sectorial agreement present larger and stronger positive abnormal returns significantly different from zero for bootstrap. For example, the complete sample in the event window presented an abnormal profitability of + 0.29%. In the case of the subsample of companies covered by the sector agreement, profitability amounts to + 0.48%, whereas the subsample of companies with their own agreement does not present any significant results.

For the 2011 reform, both subsamples behave in a similar way. We remember that the market as a whole interprets this reform as bad news. In the event window, the complete sample presents an average abnormal profitability of -4.87%, the subsample of companies with their own agreement -4.03% and the subsample of companies with a sector agreement -5%, all significant for bootstrap. The same happens with the rest of the windows, in this reform all companies, regardless of the type of agreement which they have, react negatively. We understand that, for companies with a sectorial agreement, the 2011 reform may be an incentive to abandon the sectorial agreement and agree to other conditions in their own or firm level agreement, with the loss of wealth that this type of agreement can entail for the investor.

Regarding the 2012 reform, both types of companies still do not present significant abnormal returns throughout the study period.

## 4. Sensitivity analysis

In order to verify the effects that the macroeconomic situation as a whole, as well as other characteristics of the company, may have on returns, we have incorporated all the macroeconomic variables into the market model in addition to creating a variable that controls the sector that the company belongs to at the same time. The industries are: trade and other services, other manufacturing industries, cement, glass and construction materials, finance, transport and communications, utilities and construction, among others.

*Industry* is included as dummy variable per industry, the criterion for the classification of firms follows the CNAE (National classification of economic activities), two digits, as has been shown in S1 Table. *Eventwindow* is a dummy variable that has the value 1 for the 11 days of the study window, and another dummy variable, *Agreement*, that takes the value 1 if the company has its own agreement and 0 if it has a sectorial agreement.


$$R_{it}=\alpha_{i}+\beta_{1i}Rmt+\beta_{2i}PR_{}+\beta_{3i}PC_{}+\beta_{4i}TED+\sum \beta_{k}\;Industry_{i}+\lambda \;Eventwindow_{i}+\xi Agreement_{i}+\varepsilon_{it}$$ (5)

Since a preliminary analysis of the correlation matrix of variables shows multicollinearity, this problem is solved using a Decision Tree. This technique is the graphical representation of the priority of values that certain variables take in relation to the target variable.

Although this is a new statistical method in the accounting discipline, it has been used in other fields of scientific research [22–25]. It also has several characteristics which make it very suitable for the set of variables used in this study and the objective pursued. This method prevents the use of other models, such as multiple regression models, where the target variable must follow a normal distribution, residuals must be independent and variance constant, which is not met in this study. This method also presents an excellent performance in prediction and classification tasks, comparable to support vector machines. It is used to discover patterns in data. Patterns are gathered and organised into models that are later used to establish relations of dependence between variables, thereby permitting a comparison between the impairment method and the amortisation method. Apart from this, it shows good predictive behaviour, even when the majority of the variables are noisy, and, moreover, it does not require a pre-selection of the variable; that is to say, it shows a strong robustness with respect to the set of characteristics. It can work with a mix of categorical and continuous explanatory variables. Finally, it includes interactions between explanatory variables and, besides returning important measures of the objective variable, it establishes an order of importance between the explanatory variables.

**Fig 1:**
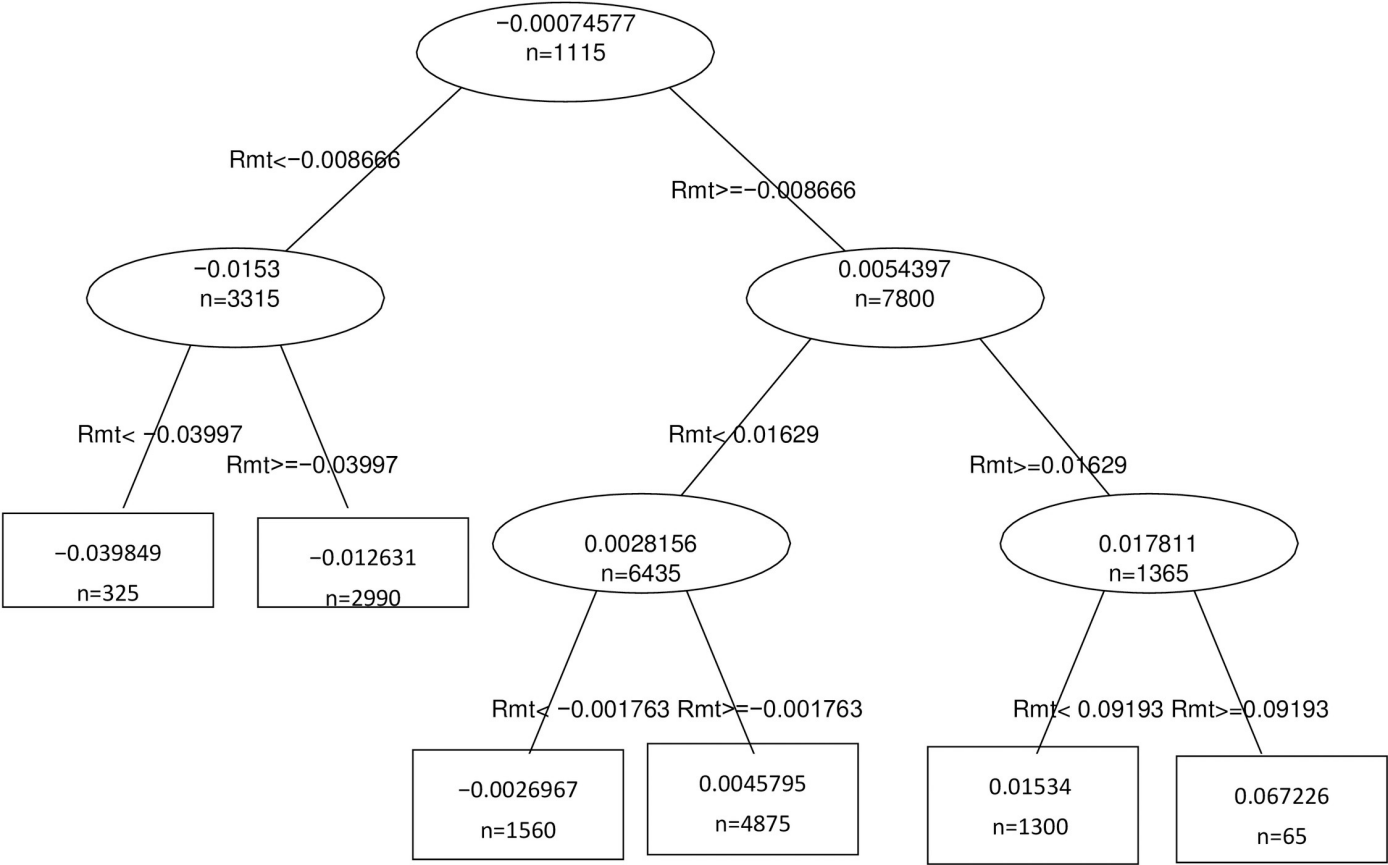
Decision Tree Reform 2010. R_it_ = α_i_+β_1i_Rmt+β_2i_PR+ β_3i_PC+β_4i_TED + Σβ_k_ Industry_i_ + +λ Eventwindow_i_ + ξAgreement _i+ εit_ *where R*_*it*_ *is the return on company i on day t*, *Rmt is the return on the market on day t (IBEX35)*, *PR Risk Premium*, *PC Slope of the Sovereign Yield Curve*, *TED Ted Spread*, *Industry is a dummy variable per industry*, *Eventwindow is a dummy variable that has the value 1 for the 11 days of the study window*, *and dummy Agreement*, *that takes the value 1 if the company has firm level agreement and 0 if it has a sectorial agreement*.

**Fig 2:**
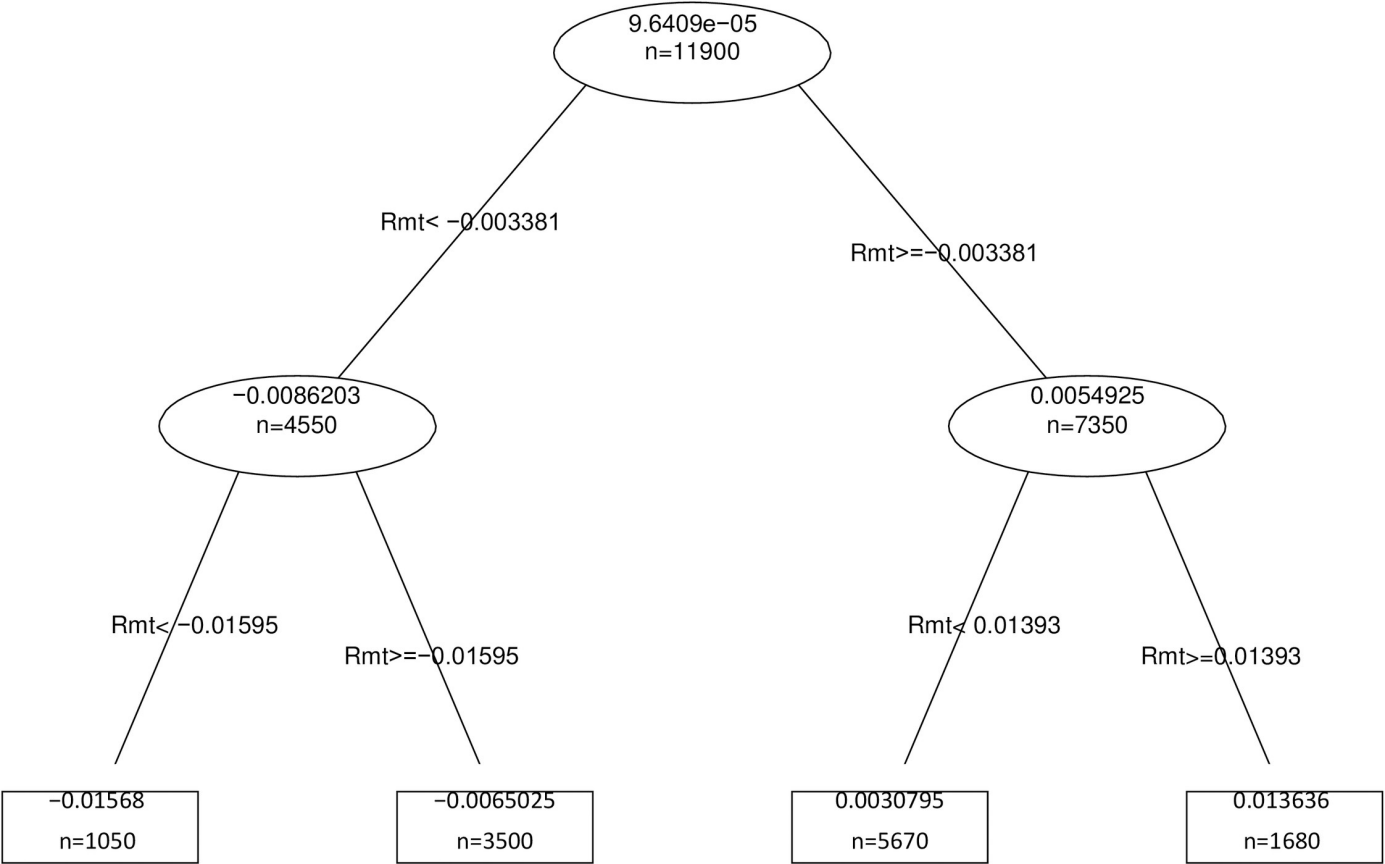
Decision Tree Reform 2011. R_it_ = α_i_+β_1i_Rmt+β_2i_PR+ β_3i_PC+β_4i_TED + Σβ_k_ Industry_i_ + +λ Eventwindow_i_ + ξAgreement _i+ εit_ *where R*_*it*_, *is the return on company i on day t*, *Rmt is the return on the market on day t (IBEX35)*, *PR Risk Premium*, *PC Slope of the Sovereign Yield Curve*, *TED Ted Spread*, *Industry is a dummy variable per industry*, *Event-window is a dummy variable that has the value 1 for the 11 days of the study window*, *and dummy Agreement*, *that takes the value 1 if the company has firm level agreement and 0 if it has a sectorial agreement*.

**Fig 3:**
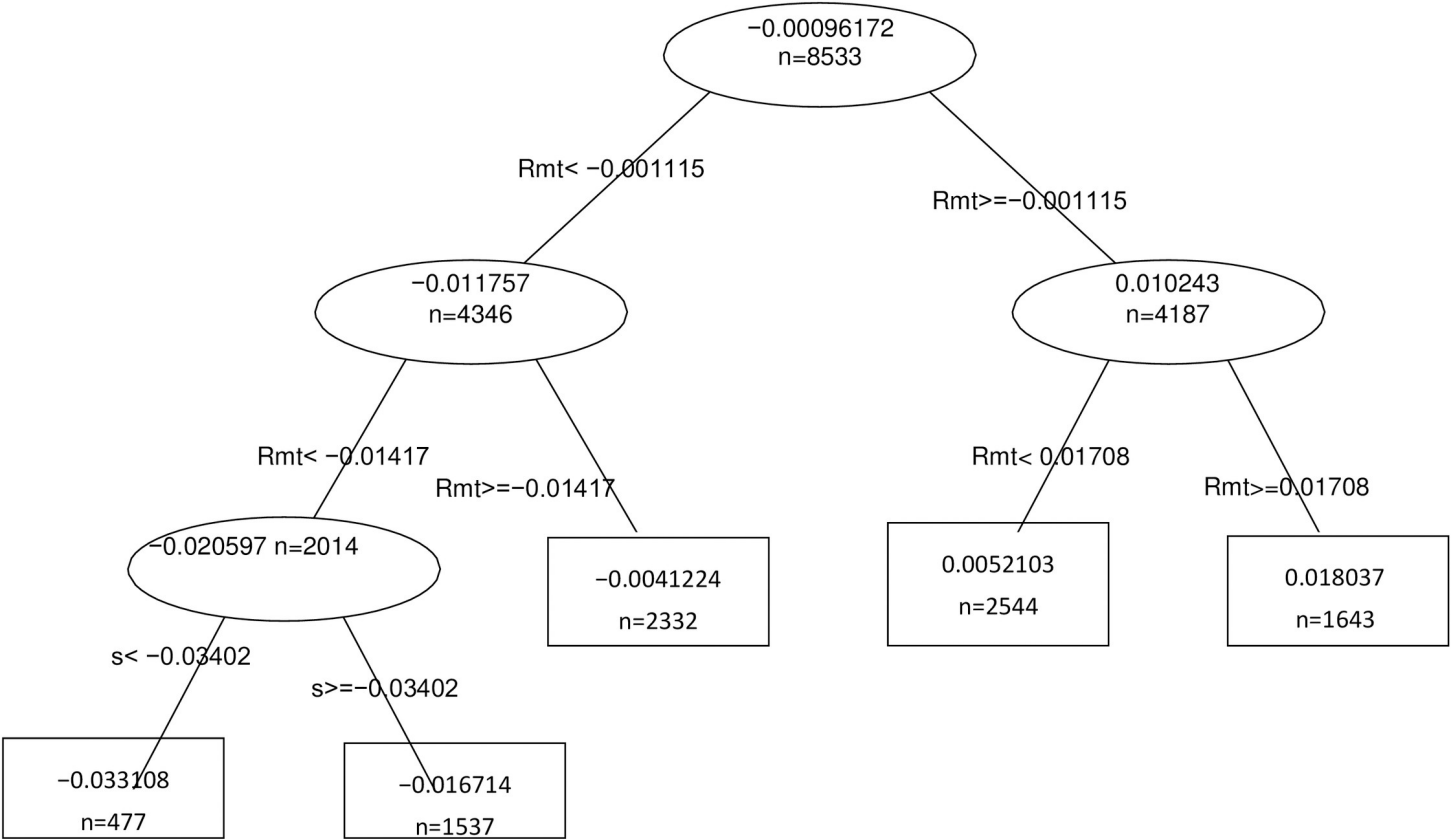
Decision Tree Reform 2012. R_it_ = α_i_+β_1i_Rmt+β_2i_PR+ β_3i_PC+β_4i_TED + Σβ_k_ Industry_i_ + +λ Eventwindow_i_ + ξAgreement _i+ εit_ *where R*_*it*_ *is the return on company i on day t*, *R*_*mt*_ *is the return on the market on day t (IBEX35)*, *PR Risk Premium*, *PC Slope of the Sovereign Yield Curve*, *TED Ted Spread*, *Industry is a dummy variable per industry*, *Eventwindow is a dummy variable that has the value 1 for the 11 days of the study window*, *and dummy Agreement*, *that takes the value 1 if the company has firm level agreement and 0 if it has a sectorial agreement*.

Figs 1–3 show the decision trees for the reforms of 2010, 2011 and 2012 respectively.

In all of them we can see that the only relevant variable when determining the reform’s influence on daily returns is the market return IBEX35. This indicates that neither the sector to which the company belongs, nor the type of agreement, nor the macroeconomic situation determines the behavior of our returns.

## 5. Conclusions

After the reforms of 2010, 2011 and 2012, others have taken place, such as the Royal Decree-Law 16/2013 of 20 December (BOE 21-12-2013), of measures to favour stable contracting and improve the workers’ employability, which contains significant changes for the workers at all levels. The entry into force on Monday 23 December 2013 marked new rules of the game in labour relations, with important changes, especially in part-time contracts and training and learning. There have also been the Royal Decree-Law 3/2014, of 28 February, of urgent measures for the promotion of employment and indefinite contracting, and the Royal Decree-Law 4/2015, of 22 March, for the urgent reform of the System of Professional Training for Employment in the labour area.

As can be observed, the Spanish work market has experienced a huge amount of reforms since 2010. Employment has not been generated but the labour reform has fulfilled part of its objectives, especially those related with giving firms tools to “ease” their labour costs through measures such as wage reductions, reductions of work time, of compensation for dismissal and of mobility of workers, be it functional or geographic.

Since the entry into force of the labour reform, wages have been reduced by 10% on average according to the results of the third Monitoring Observatory of this reform, coming from 200 surveys of firms, mainly with more than 50 employees. Another of the effects of the labour reform which is being seen, according to those in charge of the report, is the reduction in the compensation which firms pay for dismissals. Nonetheless, the information contributed only refers to collective dismissals; 24% of the total. In this case, the average compensation has been reduced to 26 days per year worked. Temporary contracts are more common than indefinite contracts and firm agreements predominate over sectorial agreements.

The idiosyncrasy of Collective Bargaining in Spain is an aspect that analysts and investors take into account due to the multitude of scenarios that, in one way or another, affect the company’s future cash flows and, therefore, modify the distribution of wealth. The labour reforms have informative content for the Spanish market, as is shown by the presence of significant abnormal returns when the information on the reform reaches the market, but the sign and the magnitude depend on its content.

The result of the labor reforms carried out between 2010 and 2012 have meant greater job destruction, greater precariousness, cheaper layoffs, and of course diminishing the power of the unions. The result of the 2010 Reform only confirms the trend of the labor market in recent decades, that is, given the news of lower labor costs, the investor interprets higher future cash flows.

It is intuitive to think that a person who is willing to work at a lower salary might find a job. However, the idea that reducing wages will eliminate unemployment is incorrect, as it is based on a classic compositional fallacy, just as the market could experience with the 2010 reform. By reducing the labor force to the status of any commodity, the false deduction is incurred that what is true for a person seeking employment is also true for all workers taken as a whole.

Business sales depend on workers having enough income to purchase the goods and services they offer, and wages are the main source of wealth for people. By reducing wage costs, entrepreneurs will find no demand for their products. Thus, a reduction in wages can worsen the profit rate and lead to a reduction in investment and worsening unemployment, we are talking about "the paradox of costs". Hence, in the face of the threat of having their wealth diminished, the shareholder reacts negatively to the 2011 Reform.

The 2012 Reform was the most radical in 30 years and the trade unions replied accordingly. In terms of collective agreements and wages, the trade unions were led to a position of defensive bargaining while the attitude of firms at the bargaining table was changed. Spain is in the top-6 countries in EU in terms of industrial action and prior literature documents an adverse relation between strikes and productivity. In particular, the announcement of the reform immediately triggered a reaction from the trade unions. Attendance at the protests was very significant, which the trade unions described as a show of ‘massive support’ from the people, and they announced an escalation of the mobilisation if the government did not improve its offer. These events, as well as the announcement of a general strike, occurred within the event window of the study and it can be argued that they would have an effect to investors, creating uncertainty in the market and contributing to potentially contradicting observations that led to the statistically insignificant result.

## Supporting information

S1 Table Sample by sector and year. (DOCX)

S2 Table Covariance correlation macro variables. (DOCX)

S3 Table Abnormal daily returns. (DOCX)

S4 Table Cumulative abnormal returns. (DOCX)

S5 Table Daily abnormal return and cumulative abnormal return by subsamples. (DOCX)

S1 Data (RAR)

## References

[1] Unionization and profitability: Evidence from the capital market. Ruback, Zimmerman R. y M.B.;. 1984, Journal of Political Economy.

[2] Unionization and profitability: Evidence of spillover effects. Bronars S. y Deere R. 1994, Journal of Political Economy, págs. Vol. 106 (6): 1281–1287.

[3] The effect of wage bargaining on the stock market value of the firm. Abowd J.M. 1989, American Economic Review, págs. Vol. 79: 774–800.

[4] Tobin´s q uninization and the concentration-profits relationship. Salinger M 1984, Journal of Economics, págs. Vol 15:159–170.

[5] Union rent seeking intangible capital and market value of the firm. Conolly R., Hirsch B. y Hirschey M. 1986, Review of Economics and Statistics, págs. Vol 38: 567–577.

[6] Unionization and firm performance: the impact on profits, growth and productivity. Clark K. 1984, American Economic Review, págs. Vol. 74: 893–919.

[7] ¿Internaliza el mercado bursátil español las relaciones laborales?: Evidencia empírica a partir de un Event Day Study. Inurrieta, A. 1997, Mimeo.

[8] ¿Observa el Mercado Español las relaciones laborales entre empresarios y sindicatos?: Un análisis empírico para el Mercado Continuo. Sabater Ana María y Laffarga Joaquina. 2006, Revista Española de Financiación y Contabilidad, págs. Vol. 128: 57–86.

[9] An empirical analysis of labor agreements on Spanish Stock Market. Sabater Ana y Laffarga Joaquina. 2011, Investment Management and Financial Innovation.

[10] Los convenios colectivos y la cotización a corto plazo de las empresas en la bolsa española. Gutiérrez Carlos y Sabater Ana. 2012, Trimestre Económico, pág. Vol. 79.

[11] Labor unions and productivity: An empirical analysis using Japanese firm-level data. Morikawa M. 2010, Labour Economics, págs. 17(6): 1030–1037.

[12] Unions and Productivity, Financial Performance and Investment: International Evidence. Metcalf D. 2002, Centre of Economic Performance Discussion Paper, págs. 1–65.

[13] Screening large-scale association study data: exploiting interactions using random forest. Lehndorff S., Dribbusch H. and Schulten T. 2018, BMC Genetics, pág. Vol. 32. 10.1186/1471-2156-5-32 PMC545646 15588316

[14] Impact of Labor Strikes on Equity Values: Canadian Evidence,. Nelson M., Amoako-Adu B. and Smith B. 1994, Journal of Economics and Business, págs. 46(3): 153–165.

[15] Kothari S. P., and Warner J.B. Econometrics of Event Studies. Handbook of Corporate Finance. Amsterdam: B. E. Eckbo, 2007.

[16] Campbell J.Y., Lo A.W., and A. Craig MacKinlay. Event-Study Analysis. The Econometrics of Financial Markets. s.l.: Princeton University Press, 1997.

[17] OECD, Statistics. https://stats.oecd.org/index.aspx?DataSetCode=CBC. [En línea] 05 de 06 de 2021. https://stats.oecd.org/index.aspx?DataSetCode=CBC.

[18] European Trade Union, Institute. Benchmarking Working Europe 2019. Brussels: ETUI Publications, 2019.

[19] European, Comission. Council Recommendation on the National Reform Programme 2011 of Spain and delivering a Council opinion on the updated Stability Programme of Spain 2011–2014,. Brussels: SEC, 2011.

[20] Vaughan-Whitehead D. Reducing Inequalities in Europe: How Industrial Relations and Labour Policies Can Close the Gap,. Cheltenham, UK: Edward Elgar Publishing, 2018.

[21] Spanish trade unions against labour market reforms: strategic choices and outcome. Del Rio Loira P. and Fenger M. 2019, 25(4), Transfer, págs. 421–435. doi: 10.1177/1024258918818267

[22] Screening large-scale association study data: exploiting interactions using random forests. Lunetta, L.B., y otros. 2004, BMC Genetics. doi: 10.1186/1471-2156-5-32 15588316 PMC545646

[23] Mining the customer credit using classification and regression tree and multivariate adaptive regresion splines. Lee T.S., y otros. 2006, Computational Statistics and Data Analysis, págs. Vol. 50: 1113–1130.

[24] Amorós A. Efectos de la adopción de la NIIF 3 en la información financiera: un estudio comparativo pre y post NIIF 3. s.l.: Tesis doctoral UMH, 2015.

[25] Big Data techniques to measure credit bankint risk in home equity loans. Pérez Martín A, Pérez Torregrosa A. y Vaca M. 2020, Journal of Business Research.

