# Supplementary material for: The influence that Spanish Labour Reform represents on Madrid Stock Market: An empirical analysis
Source: PLoS One. 2021 Oct 6;16(10):e0258004. doi: 10.1371/journal.pone.0258004 (PMC8494317; doi:10.1371/journal.pone.0258004)
Supplement: S1 Table — (DOCX) [file pone.0258004.s001.docx]

| Table 1. *Sample by sector and year* |  |  |  | **Sample 2010 N=61** | | **Sample 2011 N=70** | | **Sample 2012 N=53** | |
| --- | --- | --- | --- | --- | --- | --- | --- | --- | --- |
| **SECTOR** |  |  |  | **COLLECTIVE AGREEMENT** | **SECTOR AGREEMENT** | **COLLECTIVE AGREEMENT** | **SECTOR AGREEMENT** | **COLLECTIVE AGREEMENT** | **SECTOR AGREEMENT** |
| FOOD INDUSTRY | |  |  |  | 1 |  |  |  | 2 |
| BEVERAGE MANUFACTURING | |  |  |  | 2 |  | 1 |  |  |
| PAPER INDUSTRY |  |  |  |  | 1 |  | 2 |  | 2 |
| CHEMICAL INDUSTRY | |  |  |  | 1 |  | 1 |  | 1 |
| PHARMACEUTICAL PRODUCT MANFACTURING | | |  |  | 2 |  | 3 |  | 2 |
| NON-METALLIC MINERAL PRODUCT MANUFACTURING | | |  |  | 2 |  | 2 |  | 1 |
| METALLURGY |  |  |  | 1 | 2 | 1 | 2 |  | 2 |
| MACHINERY AND EQUIPMENT MANUF. | |  |  | 1 | 1 | 1 | 1 |  | 1 |
| OTHER MATERIAL MANUF. | |  |  | 1 | 1 | 1 | 1 | 1 | 1 |
| OTHER MANUFACTURING INDUSTRIES | | |  |  | 1 |  | 2 |  | 1 |
| SUPPLY ELECTRIC AND GAS ENERGY | | |  | 1 | 1 | 1 | 1 |  | 1 |
| WASTE COLLECTION | |  |  |  | 1 |  | 1 |  |  |
| BUILDING CONSTRUCTION | |  |  |  | 6 |  | 4 |  | 4 |
| CIVIL ENGINEERING |  |  |  |  | 3 |  | 3 |  | 2 |
| WHOLESALE TRADE | |  |  |  | 2 |  | 1 |  | 1 |
| LODGING SERVICES | |  |  |  | 1 |  | 2 |  | 2 |
| RADIO AND TV BROADCAST | |  |  | 1 | 1 | 1 | 1 | 1 | 1 |
| COMPUTER CONSULTING AND PROGRAMMING | | |  |  |  |  | 2 |  | 2 |
| FINANCIAL SERVICES EXCEPT INSURANCE | | |  | 3 | 13 | 5 | 15 | 4 | 12 |
| AUXILIARY ACTIVITIES OF FINANCIAL PRODUCTS | | |  |  |  |  | 1 |  | 1 |
| REAL ESTATE ACTIVITIES | |  |  |  | 4 |  | 2 |  | 2 |
| LEGAL AND ACCOUNTING ACTIVITTIES | | |  |  |  |  | 1 |  |  |
| BUSINESS MANAGEMENT CONSULTING ACTIVITIES | | |  | 1 | 2 |  | 3 |  | 3 |
| ARCHITECTURE AND ENGINEERING TECHNICAL SERVICES | | |  |  | 1 |  | 2 |  | 2 |
| RESEARCH AND DEVELOPMENT | |  |  |  | 1 |  | 1 |  |  |
| OTHER PROFESSIONAL, SCIENTIFIC AND TECHNICAL ACTIVITIES | | | |  | 1 |  | 1 |  |  |
| RENTING ACTIVITIES | |  |  |  |  |  | 1 |  |  |
| SAFETY AND INVESTIGATION ACTIVITIES | | |  |  | 1 |  | 1 |  |  |
| HEATLH ACTIVITIES | |  |  |  |  |  | 1 |  | 1 |
| GAMBLING AND BETTING ACTIVITIES | | |  |  |  |  | 1 |  |  |
| **TOTAL** |  |  |  | **9** | **52** | **10** | **60** | **6** | **47** |
