# Supplementary material for: The influence that Spanish Labour Reform represents on Madrid Stock Market: An empirical analysis
Source: PLoS One. 2021 Oct 6;16(10):e0258004. doi: 10.1371/journal.pone.0258004 (PMC8494317; doi:10.1371/journal.pone.0258004)
Supplement: S2 Table — (DOCX) [file pone.0258004.s002.docx]

Table 2. *Covariance correlation macro variables*

*Index IBEX35, PC Slope of the Sovereign Yield Curve, PR* *Risk Premium and TED Ted Spread*

| **Covariance** | **2010** |  |  |  | **2011** |  |  |  | **2012** |  |  |  |
| --- | --- | --- | --- | --- | --- | --- | --- | --- | --- | --- | --- | --- |
| **Correlation** |  |  |  |  |  |  |  |  |  |  |  |  |
| **t-Statistic** | **IBEX35** | **PC** | **PR** | **TED** | **IBEX35** | **PC** | **PR** | **TED** | **IBEX35** | **PC** | **PR** | **TED** |
| **IBEX35** | 0,0004 |  |  |  | 0,0002 |  |  |  | 0,0004 |  |  |  |
|  | 1,0000 |  |  |  | 1,0000 |  |  |  | 1,0000 |  |  |  |
|  | ----- |  |  |  | ----- |  |  |  | ----- |  |  |  |
|  |  |  |  |  |  |  |  |  |  |  |  |  |
| **PC** | -0,0050 | 5,4974 |  |  | 0,0006 | 0,3162 |  |  | -0,0016 | 3,7019 |  |  |
|  | -0,1009 | 1,0000 |  |  | 0,0832 | 1,0000 |  |  | -0,0410 | 1,0000 |  |  |
|  | -10,5000 | ----- |  |  | 8,6013 | ----- |  |  | -3,7433 | ----- |  |  |
|  |  |  |  |  |  |  |  |  |  |  |  |  |
| **PR** | 4.07E-05 | -0,4406 | 0,0464 |  | -0,0002 | 0,0106 | 0,0135 |  | -0,0014 | -0,0886 | 0,0980 |  |
|  | 0,0090 | -0,8725 | 1,0000 |  | -0,1112 | 0,1621 | 1,0000 |  | -0,2272 | -0,1471 | 1,0000 |  |
|  | 0,9341 | -185,6500 | ----- |  | -11,5243 | 16,9173 | ----- |  | -21,2827 | -13,5619 | ----- |  |
|  |  |  |  |  |  |  |  |  |  |  |  |  |
| **TED** | 0,0001 | -0,2191 | 0,0193 | 0,0104 | -3.80E-05 | -0,0153 | -0,0013 | 0,0011 | 0,0001 | -0,0339 | 0,0185 | 0,0115 |
|  | 0,0542 | -0,9154 | 0,8772 | 1,0000 | -0,0861 | -0,8162 | -0,3334 | 1,0000 | 0,0651 | -0,1645 | 0,5522 | 1,0000 |
|  | 5,6400 | -236,3400 | 189,8700 | ----- | -8,8975 | -145,5090 | -36,4275 | ----- | 5,9516 | -15,2148 | 60,4102 | ----- |
