# Supplementary material for: The influence that Spanish Labour Reform represents on Madrid Stock Market: An empirical analysis
Source: PLoS One. 2021 Oct 6;16(10):e0258004. doi: 10.1371/journal.pone.0258004 (PMC8494317; doi:10.1371/journal.pone.0258004)
Supplement: S3 Table — (DOCX) [file pone.0258004.s003.docx]

Table 3

*Abnormal Daily Returns AR_it_. Bootstrap Technique.*

*AR_it_=R_it_-(a_i_+b_i_R_mt_) Where a_i_ and b_i_ are the GLS estimates obtained in the regressions R_it_=α_i_+β_1i_R_mt_+β_2i_PR + β_3i_PC +β_4i_TED + ε_it_ where R_it_ is the return on company i on day t; R_mt_ is the return on the market on day t;* *PR* *Risk Premium , PC Slope of the Sovereign Yield Curve and TED Ted Spread.*

| **Day** | **-5** | **-4** | **-3** | **-2** | **-1** | **0** | **1** | **2** | **3** | **4** | **5** |
| --- | --- | --- | --- | --- | --- | --- | --- | --- | --- | --- | --- |
| **Event 2010 N=61** |  |  |  |  |  |  |  |  |  |  |  |
| **Market Model** |  |  |  |  |  |  |  |  |  |  |  |
| **AR** | **-0.0009** | **-0.0058*** | **0.0020** | **0.0067*** | **0.0011** | **-0.0020** | **-0.0080***** | **0.0038*** | **0.0029** | **0.0069**** | **-0.0024** |
| t statistic | -0.3379 | -1.8957 | 0.6168 | 1.8857 | 0.4521 | -0.7040 | -2.9019 | 1.7209 | 0.9984 | 2.0392 | -0.9210 |
| P value | 0.7352 | 0.0734 | 0.5730 | 0.0390 | 0.6542 | 0.5230 | 0.0070 | 0.0948 | 0.3062 | 0.0120 | 0.4194 |
| **Market Model with PC** |  |  |  |  |  |  |  |  |  |  |  |
| **AR** | **0.0013** | **-0.0035** | **0.0041** | **0.0088***** | **0.0032** | **0.0002** | **-0.0057**** | **0.0062***** | **0.0053*** | **0.0093***** | **0.0001** |
| t statistic | 0.4710 | -1.1025 | 1.2278 | 2.3752 | 1.1481 | 0.0817 | -2.0585 | 2.5058 | 1.9230 | 2.7472 | 0.0308 |
| P value | 0.6482 | 0.2902 | 0.2490 | 0.0090 | 0.2486 | 0.9014 | 0.0470 | 0.0228 | 0.0332 | 0.0008 | 0.9634 |
| **Market Model with PR** |  |  |  |  |  |  |  |  |  |  |  |
| **AR** | **0.0030** | **-0.0016** | **0.0061*** | **0.0109***** | **0.0056*** | **0.0023** | **-0.0045*** | **0.0066***** | **0.0061**** | **0.0104***** | **0.0009** |
| t statistic | 1.0152 | -0.4933 | 1.6933 | 2.7459 | 1.8452 | 0.8025 | -1.6213 | 2.6724 | 2.2858 | 2.9410 | 0.3494 |
| P value | 0.3248 | 0.6438 | 0.1116 | 0.0020 | 0.0568 | 0.3998 | 0.1210 | 0.0156 | 0.0072 | 0.0002 | 0.7072 |
| **Market model with TED** |  |  |  |  |  |  |  |  |  |  |  |
| **AR** | **0.0014** | **-0.0033** | **0.0045** | **0.0091***** | **0.0033** | **0.0002** | **-0.0058**** | **0.0058**** | **0.0049*** | **0.0088***** | **-0.0005** |
| t statistic | 0.4713 | -0.9832 | 1.2577 | 2.3306 | 1.1781 | 0.0824 | -2.0722 | 2.2859 | 1.7670 | 2.5919 | -0.1872 |
| P value | 0.6484 | 0.3424 | 0.2320 | 0.0114 | 0.2284 | 0.9354 | 0.0372 | 0.0314 | 0.0576 | 0.0008 | 0.8642 |
|  |  |  |  |  |  |  |  |  |  |  |  |

| **Day** | **-5** | **-4** | **-3** | **-2** | **-1** | **0** | **1** | **2** | **3** | **4** | **5** |
| --- | --- | --- | --- | --- | --- | --- | --- | --- | --- | --- | --- |
| **Event 2011 N=70** |  |  |  |  |  |  |  |  |  |  |  |
| **Market Model** |  |  |  |  |  |  |  |  |  |  |  |
| **AR** | **-0.0052***** | **-0.0017** | **-0.0071***** | **0.0002** | **-0.0087***** | **-0.0062***** | **0.0015** | **0.0021** | **-0.0070***** | **-0.0094***** | **-0.0091***** |
| t statistic | -3.4702 | -1.0127 | -2.9524 | 0.0943 | -4.4791 | -2.7325 | 0.6690 | 1.0574 | -2.8934 | -3.8150 | -4.4999 |
| P value | 0.0002 | 0.2890 | 0.0020 | 0.9192 | 0.0000 | 0.0032 | 0.4644 | 0.2630 | 0.0060 | 0.0000 | 0.0000 |
| **Market Model with PC** |  |  |  |  |  |  |  |  |  |  |  |
| **AR** | **-0.0050***** | **-0.0015** | **-0.0068***** | **0.0005** | **-0.0085***** | **-0.0059***** | **0.0018** | **0.0023** | **-0.0067***** | **-0.0090***** | **-0.0087***** |
| t statistic | -3.2443 | -0.8525 | -2.8565 | 0.2446 | -4.4370 | -2.6285 | 0.7820 | 1.1955 | -2.7712 | -3.7144 | -4.2340 |
| P value | 0.0010 | 0.3772 | 0.0018 | 0.8146 | 0.0000 | 0.0054 | 0.3902 | 0.2162 | 0.0100 | 0.0000 | 0.0000 |
| **Market Model with PR** |  |  |  |  |  |  |  |  |  |  |  |
| **AR** | **-0.0050***** | **-0.0016** | **-0.0067***** | **0.0011** | **-0.0073***** | **-0.0045**** | **0.0028** | **0.0038*** | **-0.0044*** | **-0.0073***** | **-0.0072***** |
| t statistic | -3.3615 | -0.9620 | -2.7641 | 0.5894 | -3.7402 | -2.0438 | 1.2033 | 1.9580 | -1.8807 | -2.8474 | -3.5317 |
| P value | 0.0008 | 0.3174 | 0.0040 | 0.5592 | 0.0004 | 0.0300 | 0.1738 | 0.0464 | 0.1026 | 0.0020 | 0.0000 |
| **Market Model with TED** |  |  |  |  |  |  |  |  |  |  |  |
| **AR** | **-0.0050***** | **-0.0016** | **-0.0070***** | **0.0003** | **-0.0087***** | **-0.0061***** | **0.0016** | **0.0021** | **-0.0069***** | **-0.0092***** | **-0.0089***** |
| t statistic | -3.2665 | -0.9398 | -2.9228 | 0.1562 | -4.4420 | -2.6881 | 0.6978 | 1.0525 | -2.8181 | -3.7892 | -4.3457 |
| P value | 0.0002 | 0.3342 | 0.0030 | 0.8550 | 0.0000 | 0.0022 | 0.4572 | 0.2784 | 0.0064 | 0.0000 | 0.0000 |
|  |  |  |  |  |  |  |  |  |  |  |  |

| **Day** | **-5** | **-4** | **-3** | **-2** | **-1** | **0** | **1** | **2** | **3** | **4** | **5** |
| --- | --- | --- | --- | --- | --- | --- | --- | --- | --- | --- | --- |
| **Event 2012 N=53** |  |  |  |  |  |  |  |  |  |  |  |
| **Market Model** |  |  |  |  |  |  |  |  |  |  |  |
| **RA** | **0.0045**** | **-0.0015** | **0.0008** | **-0.0033** | **0.0039*** | **0.0024** | **-0.0001** | **-0.0020** | **-0.0007** | **-0.0021** | **0.0046** |
| t statistic | 2.3043 | -0.6625 | 0.3269 | -1.4544 | 1.7847 | 1.0578 | -0.0291 | -0.6344 | -0.2650 | -0.7447 | 0.8709 |
| P value | 0.0118 | 0.5196 | 0.8036 | 0.2012 | 0.0636 | 0.2480 | 0.9796 | 0.4966 | 0.7972 | 0.4936 | 0.2758 |
| **Market Model with PC** |  |  |  |  |  |  |  |  |  |  |  |
| **AR** | **0.0034*** | **-0.0027** | **-0.0004** | **-0.0047**** | **0.0021** | **0.0005** | **-0.0025** | **-0.0045** | **-0.0029** | **-0.0036** | **0.0022** |
| t statistic | 1.6872 | -1.1407 | -0.1507 | -2.1626 | 0.8032 | 0.1887 | -0.6238 | -1.3067 | -0.9793 | -1.3178 | 0.3853 |
| P value | 0.0752 | 0.2598 | 0.8218 | 0.0462 | 0.4230 | 0.8142 | 0.6114 | 0.1816 | 0.3422 | 0.1834 | 0.6254 |
| **Market Model with PR** |  |  |  |  |  |  |  |  |  |  |  |
| **AR** | **0.0040**** | **-0.0019** | **0.0008** | **-0.0034** | **0.0044**** | **0.0023** | **0.0003** | **-0.0014** | **-0.0006** | **-0.0013** | **0.0046** |
| t statistic | 2.0380 | -0.8371 | 0.3231 | -1.4955 | 1.9987 | 1.0330 | 0.0963 | -0.4430 | -0.2306 | -0.4502 | 0.8817 |
| P value | 0.0256 | 0.4084 | 0.7778 | 0.1896 | 0.0382 | 0.2594 | 0.8662 | 0.6562 | 0.8452 | 0.6582 | 0.2664 |
| **Market Model with TED** |  |  |  |  |  |  |  |  |  |  |  |
| **AR** | **0.0045**** | **-0.0014** | **0.0009** | **-0.0030** | **0.0039*** | **0.0026** | **0.0002** | **-0.0018** | **-0.0007** | **-0.0018** | **0.0049** |
| t statistic | 2.2736 | -0.6072 | 0.3728 | -1.3620 | 1.7919 | 1.1500 | 0.0471 | -0.5542 | -0.2469 | -0.6387 | 0.9392 |
| P value | 0.0142 | 0.5492 | 0.7584 | 0.2422 | 0.0698 | 0.1982 | 0.8960 | 0.5534 | 0.8002 | 0.5484 | 0.2344 |

**Significant at 10%. ** Significant at 5%. *** Significant at 1%.*

*Source: Own construction.*
