# Supplementary material for: The influence that Spanish Labour Reform represents on Madrid Stock Market: An empirical analysis
Source: PLoS One. 2021 Oct 6;16(10):e0258004. doi: 10.1371/journal.pone.0258004 (PMC8494317; doi:10.1371/journal.pone.0258004)
Supplement: S4 Table — (DOCX) [file pone.0258004.s004.docx]

Table 4

*Cumulative Abnormal Returns. CAR. Bootstrap Technique.*

*CAR=∑AR_it_ ; AR_it_=R_it_-(a_i_+b_i_R_mt_) Where a_i_ and b_i_ are the GLS estimates obtained in the regressions R_it_=α_i_+β_1i_R_mt_+β_2i_PR + β_3i_PC +β_4i_TED + ε_it_ where R_it_ is the return on company i on day t; R_mt_ is the return on the market on day t; PR Risk Premium , PC Slope of the Sovereign Yield Curve and TED Ted Spread.*

| **Window** | **(-5,+5)** | **(-3,+3)** | **(-2,+2)** | **(-1,+1)** | **(-5,-1)** | **(+1,+5)** |
| --- | --- | --- | --- | --- | --- | --- |
| **Event 2010 N=61** | |  |  |  |  |  |
| **Market Model** | |  |  |  |  |  |
| **CAR** | **0.0044** | **0.0065** | **0.0016** | **-0.0089***** | **0.0032** | **0.0032** |
| t statistic | 0.6245 | 1.4803 | 0.3885 | -2.7591 | 0.4861 | 0.6029 |
| P value | 0.5170 | 0.1218 | 0.6846 | 0.0098 | 0.6228 | 0.5376 |
| **Market Model with PC** | | |  |  |  |  |
| **CAR** | **0.0294***** | **0.0222***** | **0.0128**** | **-0.0022** | **0.0140*** | **0.0152***** |
| t statistic | 2.7553 | 3.1719 | 2.1036 | -0.5816 | 1.7071 | 2.6907 |
| P value | 0.0104 | 0.0014 | 0.0376 | 0.5516 | 0.0994 | 0.0084 |
| **Market Model with PR** | | |  |  |  |  |
| **CAR** | **0.0458***** | **0.0331***** | **0.0209***** | **0.0034** | **0.0240***** | **0.0195***** |
| t statistic | 3.4477 | 3.8321 | 2.8779 | 0.7991 | 2.4175 | 3.2883 |
| P value | 0.0004 | 0.0000 | 0.0028 | 0.4232 | 0.0126 | 0.0018 |
| **Market model with TED** | | |  |  |  |  |
| **CAR** | **0.0285**** | **0.0221***** | **0.0127*** | **-0.0022** | **0.0151** | **0.0132**** |
| t statistic | 2.2082 | 2.6218 | 1.8568 | -0.5704 | 1.5469 | 2.2354 |
| P value | 0.0254 | 0.0054 | 0.0598 | 0.5584 | 0.1238 | 0.0258 |
|  |  |  |  |  |  |  |

| **Event 2011 N=70** | |  |  |  |  |  |
| --- | --- | --- | --- | --- | --- | --- |
| **Market Model** | |  |  |  |  |  |
| **Window** | **(-5,+5)** | **(-3,+3)** | **(-2,+2)** | **(-1,+1)** | **(-5,-1)** | **(+1,+5)** |
| **CAR** | **-0.0506***** | **-0.0252***** | **-0.0111**** | **-0.0134***** | **-0.0226***** | **-0.0218***** |
| t statistic | -7.3239 | -4.2671 | -2.1892 | -3.2915 | -6.0297 | -5.7581 |
| P value | 0.0000 | 0.0000 | 0.0158 | 0.0014 | 0.0000 | 0.0000 |
| **Market Model with PC** | | |  |  |  |  |
| **CAR** | **-0.0474***** | **-0.0233***** | **-0.0097*** | **-0.0125***** | **-0.0212***** | **-0.0203***** |
| t statistic | -6.7410 | -3.9248 | -1.9066 | -3.0870 | -5.7148 | -5.0873 |
| P value | 0.0000 | 0.0000 | 0.0470 | 0.0022 | 0.0000 | 0.0000 |
| **Market Model with PR** | | |  |  |  |  |
| **CAR** | **-0.0363***** | **-0.0152***** | **-0.0041** | **-0.0091**** | **-0.0195***** | **-0.0123***** |
| t statistic | -5.2577 | -2.5529 | -0.7963 | -2.2026 | -5.3003 | -2.9903 |
| P value | 0.0000 | 0.0114 | 0.4186 | 0.0278 | 0.0000 | 0.0038 |
| **Market model with TED** | | |  |  |  |  |
| **CAR** | **-0.0494***** | **-0.0247***** | **-0.0108**** | **-0.0132***** | **-0.0220***** | **-0.0213***** |
| t statistic | -6.9780 | -4.1019 | -2.1016 | -3.2014 | -5.8326 | -5.4587 |
| P value | 0.0000 | 0.0000 | 0.0246 | 0.0004 | 0.0000 | 0.0000 |
|  |  |  |  |  |  |  |
| **Event 2012 N=53** | |  |  |  |  |  |
| **Market Model** | |  |  |  |  |  |
| **Window** | **(-5,+5)** | **(-3,+3)** | **(-2,+2)** | **(-1,+1)** | **(-5,-1)** | **(+1,+5)** |
| **CAR** | **0.0063** | **0.0009** | **0.0008** | **0.0061** | **0.0044** | **-0.0004** |
| t statistic | 0.7273 | 0.1633 | 0.1512 | 1.1862 | 0.8950 | -0.0585 |
| P value | 0.4500 | 0.8954 | 0.8830 | 0.1802 | 0.3516 | 0.9934 |
| **Market Model with PC** | | |  |  |  |  |
| **CAR** | **-0.0130** | **-0.0123** | **-0.0090** | **0.0001** | **-0.0022** | **-0.0113** |
| t statistic | -0.8692 | -1.2389 | -1.0401 | 0.0172 | -0.3579 | -1.1548 |
| P value | 0.4200 | 0.2234 | 0.2950 | 0.9942 | 0.7268 | 0.3142 |
| **Market Model with PR** | | |  |  |  |  |
| **CAR** | **0.0078** | **0.0024** | **0.0023** | **0.0071** | **0.0039** | **0.0016** |
| t statistic | 0.8925 | 0.4401 | 0.4029 | 1.3555 | 0.7975 | 0.2229 |
| P value | 0.3632 | 0.6840 | 0.6874 | 0.1318 | 0.4230 | 0.7996 |
| **Market model with TED** | | |  |  |  |  |
| **CAR** | **0.0083** | **0.0020** | **0.0018** | **0.0067** | **0.0048** | **0.0008** |
| t statistic | 0.9327 | 0.3778 | 0.3287 | 1.2792 | 0.9727 | 0.1156 |
| P value | 0.3350 | 0.7224 | 0.7484 | 0.1538 | 0.3246 | 0.8816 |

**Significant at 10%. ** Significant at 5%. *** Significant at 1%.*

*Source: Own construction.*
