# Supplementary material for: The influence that Spanish Labour Reform represents on Madrid Stock Market: An empirical analysis
Source: PLoS One. 2021 Oct 6;16(10):e0258004. doi: 10.1371/journal.pone.0258004 (PMC8494317; doi:10.1371/journal.pone.0258004)
Supplement: S5 Table — (DOCX) [file pone.0258004.s005.docx]

| **Day/ windofigurew** | **-5** | **-4** | **-3** | **-2** | **-1** | **0** | **1** | **2** | **3** | **4** | **5** | **(-5,+5)** | **(-3,+3)** | **(-2,+2)** | **(-1,+1)** | **(-5,-1)** | **(+1,+5)** |
| --- | --- | --- | --- | --- | --- | --- | --- | --- | --- | --- | --- | --- | --- | --- | --- | --- | --- |
| **RA/CAR** | **Event 2010 N=61** |  |  |  |  |  |  |  |  |  |  |  |  |  |  |  |  |
| **Total sample** | 0.0013 | -0.0035 | 0.0041 | 0.0088 | 0.0032 | 0.0002 | -0.0057 | 0.0062 | 0.0053 | 0.0093 | 0.0001 | 0.0294 | 0.0222 | 0.0128 | -0.0022 | 0.0140 | 0.0152 |
| **t statistic** | 0.4710 | -1.1025 | 1.2278 | 2.3752 | 1.1481 | 0.0817 | -2.0585 | 2.5058 | 1.9230 | 2.7472 | 0.0308 | 2.7553 | 3.1719 | 2.1036 | -0.5816 | 1.7071 | 2.6907 |
| **p-value** | 0.6482 | 0.2902 | 0.249 | 0.009 | 0.2486 | 0.9014 | 0.047 | 0.0228 | 0.0332 | 0.0008 | 0.9634 | 0.0104 | 0.0014 | 0.0376 | 0.5516 | 0.0994 | 0.0084 |
| **Agreement subsample** | -0.0021 | -0.0123 | 0.0133 | 0.0068 | 0.0016 | -0.0101 | 0.0006 | 0.0027 | -0.0056 | -0.0017 | -0.0076 | -0.0143 | 0.0094 | 0.0017 | -0.0078 | 0.0073 | -0.0116 |
| **t statistic** | -0.3435 | -1.9062 | 1.6514 | 0.9142 | 0.3880 | -2.8690 | 0.1056 | 0.3706 | -1.2291 | -0.5311 | -2.2356 | -0.7218 | 0.6205 | 0.1152 | -0.9902 | 0.5295 | -1.3341 |
| **p-value** | 0.7126 | 0.0702 | 0.073 | 0.2544 | 0.7786 | 0.0324 | 0.8384 | 0.6706 | 0.1164 | 0.6006 | 0.0308 | 0.5918 | 0.5186 | 0.9028 | 0.2994 | 0.5348 | 0.1944 |
| **Sector subsample** | 0.0041 | 0.0021 | 0.0077 | 0.0042 | 0.0067 | 0.0018 | -0.0022 | 0.0034 | 0.0075 | 0.0096 | 0.0033 | 0.0483 | 0.0291 | 0.0139 | 0.0063 | 0.0249 | 0.0216 |
| **t statistic** | 1.5872 | 0.5736 | 2.7302 | 1.4192 | 1.6739 | 0.6580 | -0.7067 | 1.5497 | 2.5543 | 3.2609 | 1.5474 | 4.5691 | 3.5473 | 2.1471 | 1.0880 | 3.1935 | 4.3472 |
| **p-value** | 0.1100 | 0.5404 | 0.0116 | 0.1256 | 0.0298 | 0.4604 | 0.5312 | 0.1382 | 0.0036 | 0 | 0.1068 | 0 | 0 | 0.0188 | 0.218 | 0.0014 | 0 |
|  | **Event 2011 N=70** |  |  |  |  |  |  |  |  |  |  |  |  |  |  |  |  |
| **Total sample** | -0.0052 | -0.0018 | -0.0072 | 0.0006 | -0.0081 | -0.0071 | 0.0021 | 0.0020 | -0.0062 | -0.0092 | -0.0086 | -0.0487 | -0.0239 | -0.0105 | -0.0131 | -0.0217 | -0.0200 |
| **t statistic** | -3.5095 | -1.0612 | -3.1061 | 0.3282 | -4.3943 | -2.7015 | 0.9022 | 1.0441 | -2.6423 | -3.9353 | -4.3527 | -6.9905 | -4.1325 | -2.0926 | -3.2347 | -5.9551 | -5.1010 |
| **p-value** | 0.0004 | 0.2658 | 0.0006 | 0.7378 | 0 | 0.0022 | 0.3274 | 0.278 | 0.009 | 0 | 0 | 0 | 0 | 0.0284 | 0.0006 | 0 | 0 |
| **Agreement subsample** | 0.0018 | 0.0001 | -0.0052 | 0.0065 | -0.0085 | -0.0080 | -0.0018 | 0.0086 | -0.0122 | -0.0089 | -0.0128 | -0.0403 | -0.0206 | -0.0031 | -0.0183 | -0.0053 | -0.0270 |
| **t statistic** | 0.6015 | 0.0255 | -1.5625 | 2.7584 | -1.6711 | -1.6916 | -0.4221 | 1.4080 | -1.6556 | -3.1611 | -2.0613 | -1.5746 | -0.9905 | -0.2103 | -1.9443 | -0.4959 | -1.9598 |
| **p-value** | 0.5556 | 0.963 | 0.193 | 0.0266 | 0.1062 | 0.0358 | 0.6874 | 0.113 | 0.0476 | 0.0138 | 0.0068 | 0.0944 | 0.2758 | 0.8082 | 0.043 | 0.625 | 0.0476 |
| **Sector subsample** | -0.0064 | -0.0021 | -0.0075 | -0.0004 | -0.0081 | -0.0069 | 0.0027 | 0.0009 | -0.0052 | -0.0092 | -0.0079 | -0.0501 | -0.0245 | -0.0118 | -0.0122 | -0.0244 | -0.0188 |
| **t statistic** | -3.9426 | -1.1338 | -2.8381 | -0.1806 | -4.0282 | -2.3342 | 1.0520 | 0.4450 | -2.1205 | -3.4344 | -3.8151 | -7.1321 | -4.1553 | -2.1927 | -2.7337 | -6.4499 | -4.7113 |
| **p-value** | 0.0002 | 0.2474 | 0.002 | 0.86 | 0.0002 | 0.0068 | 0.2394 | 0.6296 | 0.0756 | 0 | 0 | 0 | 0 | 0.0192 | 0.0052 | 0 | 0 |
|  | **Event 2012 N=53** |  |  |  |  |  |  |  |  |  |  |  |  |  |  |  |  |
| **Total sample** | 0.0034 | -0.0027 | -0.0004 | -0.0047 | 0.0021 | 0.0005 | -0.0025 | -0.0045 | -0.0029 | -0.0036 | 0.0022 | -0.0130 | -0.0123 | -0.0090 | 0.0001 | -0.0022 | -0.0113 |
| **t statistic** | 1.6872 | -1.1407 | -0.1507 | -2.1626 | 0.8032 | 0.1887 | -0.6238 | -1.3067 | -0.9793 | -1.3178 | 0.3853 | -0.8692 | -1.2389 | -1.0401 | 0.0172 | -0.3579 | -1.1548 |
| **p-value** | 0.0772 | 0.2538 | 0.848 | 0.0432 | 0.419 | 0.827 | 0.6104 | 0.178 | 0.3356 | 0.1792 | 0.631 | 0.4082 | 0.2192 | 0.3012 | 0.9784 | 0.7516 | 0.3156 |
| **Agreement subsample** | -0.0002 | -0.0082 | -0.0005 | -0.0100 | 0.0057 | 0.0017 | 0.0012 | -0.0032 | 0.0065 | -0.0115 | -0.0079 | -0.0264 | 0.0014 | -0.0046 | 0.0086 | -0.0132 | -0.0149 |
| **t statistic** | -0.0610 | -1.5982 | -0.0822 | -1.5610 | 1.2983 | 0.4435 | 0.1782 | -1.1977 | 5.1174 | -1.3472 | -2.0790 | -0.9195 | 0.0762 | -0.2681 | 0.7375 | -0.9838 | -1.0326 |
| **p-value** | 0.9906 | 0.1542 | 0.8794 | 0.0956 | 0.3816 | 0.7012 | 0.887 | 0.2274 | 0.0894 | 0.1138 | 0.1448 | 0.5084 | 0.9488 | 0.7494 | 0.4394 | 0.6058 | 0.3072 |
| **Sector subsample** | 0.0039 | -0.0020 | -0.0003 | -0.0040 | 0.0016 | 0.0004 | -0.0030 | -0.0046 | -0.0041 | -0.0026 | 0.0034 | -0.0113 | -0.0140 | -0.0096 | -0.0010 | -0.0008 | -0.0109 |
| **t statistic** | 1.7244 | -0.7747 | -0.1316 | -1.7341 | 0.5645 | 0.1189 | -0.6663 | -1.2036 | -1.2429 | -0.9009 | 0.5468 | -0.6834 | -1.2798 | -1.0009 | -0.1206 | -0.1204 | -0.9932 |
| **p-value** | 0.0784 | 0.4346 | 0.8698 | 0.1218 | 0.5788 | 0.8888 | 0.5822 | 0.2248 | 0.2414 | 0.3734 | 0.4806 | 0.4916 | 0.2088 | 0.3128 | 0.9164 | 0.9124 | 0.3862 |

Table. 5 DAILY ABNORMAL RETURN AND CUMULATIVE ABNORMAL RETURN BY SUBSAMPLES. *Bootstrap Technique.* *The table show the effect on the market variables for subsample with firm level agreement (agreement subsample) or sectorial agreement (sector subsample). The variables are: AR= abnormal daily returns; CAR= cumulative abnormal returns; CAR=∑AR_it_ ; AR_it_=R_it_-(a_i_+b_i_R_mt_)* ***w****here a_i_ and b_i_ are the GLS estimates obtained in the regressions R_it_=α_i_+β_1i_R_mt_+β_3i_PC + ε_it_ where R_it_ is the return on company i on day t; R_mt_ is the return on the market on day t and PC Slope of the Sovereign Yield Curve.*
